# The estimation and partitioning of evapotranspiration in a coniferous plantation in subtropical China

**DOI:** 10.3389/fpls.2023.1120202

**Published:** 2023-03-01

**Authors:** Mingjie Xu, Qianhui Ma, Shengtong Li, Fengting Yang, Tao Zhang, Fei Xu, Bin Yang, Hui Zhang, Shu Zhang, Qianyu Wang, Yuanyuan Tang, Huimin Wang

**Affiliations:** ^1^ College of Agronomy, Shenyang Agricultural University, Shenyang, China; ^2^ Key Laboratory of Ecosystem Network Observation and Modeling, Institute of Geographic Sciences and Natural Resources Research, Chinese Academy of Sciences, Beijing, China; ^3^ Key Laboratory of Tropical Forest Ecology, Xishuangbanna Tropical Botanical Garden, Chinese Academy of Sciences, Menglun, China; ^4^ Jinzhou Ecology and Agriculture Meteorological Center, Liaoning Meteorological Bureau, Jinzhou, China; ^5^ Heilongjiang Province Meteorological Service Center, Harbin, China; ^6^ University of Chinese Academy of Sciences, Beijing, China

**Keywords:** SWH model, subtropical plantation, eddy covariance, evapotranspiration, evaporation, transpiration

## Abstract

Accurate estimations of forest evapotranspiration (ET) and its components, transpiration (T) and evaporation (E), are important for deep understanding and predicting the responses of forest water cycles to climate change. In this study, the improved Shuttleworth-Wallace model (SWH) was applied to estimate ET, T, and E during 2003–2014 in a subtropical planation, and the modeled results were verified using *in situ* measurements by the eddy covariance technique, sap flow, and micro-lysimeter method. The study aimed to clarify whether it is feasible and reliable to use the SWH model to estimate and partition ET in forests. In addition, depending on the long-term data, the specific performances in modeling ET under different climatic backgrounds were investigated, and the underlying mechanisms were explored. The results verified that the SWH performed relatively well in the subtropical forest, and the modeled ET, T and E could track the seasonal variations, although overestimations were found in the peak seasons. However, the model was relatively weaker in estimating the interannual variabilities. It performed well in modeling ET in normal years but showed larger model residuals in years with obvious climatic anomalies. In the severe summer-drought (2003) and cold-spring (2005) years, the model greatly overestimated ET. It also overestimated ET in summer since 2010, which may be ascribed to the less dependency of ET on VPD induced by the more humid microclimate in forest accompanied with forest development. For the ET partitioning results, the modeled and measured E and T values were all in reasonable ranges. The possible reasons for underestimations (overestimations) of E and T by measurements (SWH model) were discussed. In this study, the data obtained using different methods and from different scales matched each other and could be cross validated, and the discussion on discrepancies would be beneficial for understanding the advantages and flaws of different methods and could be the basis for optimizing the measurement and model methods. In sum, this study verified that it is feasible to use the SWH model in forests and provided a basis for further improving and optimizing the modeled results under different climate backgrounds.

## 1 Introduction

Under climate change, warming and changing precipitation patterns would probably greatly affect the water cycle of terrestrial ecosystems (Jung et al., 2010). Evapotranspiration (ET) plays an important role in regulating the water cycle and accounts for a large proportion of annual precipitation (Wang and Dickinson, 2012; Ha et al., 2015). Forests cover approximately 30% of the global land surface and account for more than 45% of the global terrestrial ET (FAO, 2006). Besides the direct water and energy exchange accompanied by ET, forest ET plays an important role in other key ecological processes and can even regulate the local climate through its biophysical effects (Fisher et al., 2011). Therefore, accurate estimations and a deep understanding of forest ET would help improve the knowledge of water cycle processes and provide a better understanding of their ecological functions (Zhu et al., 2015; Gao et al., 2016).

Studies on ET have a long history and are a classical research field. Many methods have been developed and applied to measure and estimate ET, such as the lysimeter method (Boast and Robertson, 1982), eddy covariance (EC) technique (Oishi et al., 2010; Xu et al., 2014), classical Penman-Monteith model (Bao et al., 2021b), and remote sensing method (Li et al., 2009). In the beginning, studies on ET were widely carried out in crop fields and grasslands (Moran et al., 2009; Zhu et al., 2013), whose structures are relatively simple and spatially homogeneous. Although the ET of forests is very important in large amounts, fewer studies have been carried out in forests due to their complicated structures, which makes it difficult to observe and quantify variations in ET. Benefitting from the wide applications of the EC technique, there have been studies focused on the intra- and interannual variations in ET in recent years (Xu et al., 2014; Gao et al., 2016; Guerrieri et al., 2016). However, it is infeasible to install and maintain EC systems in overlarge areas due to the large labor and economic consumption. Therefore, the model method has advantages on a large spatial scale, and EC could provide *in situ* data to verify and modify the model estimation.

As the sum of evaporation (E) and transpiration (T), accurate estimations of each component of ET are vital for understanding the water use efficiency and underlying mechanisms of ET variations under global climate change (Lawrence et al., 2007; Hu et al., 2013; Lu et al., 2014; Nguyen et al., 2021). *In situ* observations could be used to separate ET into evaporation and transpiration. For evaporation, the lysimeter method is an easier and generally applied method (Yang et al., 2018; Gong et al., 2019). However, few studies have reported *in situ* measurements of evaporation in forests, as it is labor consuming and difficult to obtain long-term continuous data. For transpiration, the sap flow method provides the possibility of accurate estimation of forest transpiration (Tognetti et al., 2009; Kume et al., 2011; Yang et al., 2022). However, these two methods are difficult to apply on a regional scale and are infeasible on long time scales.

Models are another option for estimating and separating ET across large areas (Ortega-Farias et al., 2010; Fisher et al., 2011; Kool et al., 2014). Among the ET models, the Penman-Monteith model has a solid theoretical basis with high accuracy and has been widely used (Wang and Dickinson, 2012). The Shuttleworth-Wallace (SW) model is an improvement of the Penman-Monteith model with higher simulation accuracy (Hu et al., 2009; Li and Zhang, 2011). In addition, it is a two-source model that could be used to separate ET into evaporation and transpiration. Hu et al. (2013) improved the SW model to the SWH model and applied it in farmland and grassland ecosystems according to previous studies, and the model performed well (Hu et al., 2013; Yang et al., 2018; Bao et al., 2021a; Bao et al., 2021b). However, few studies have reported the verification and validation results of the SWH model in simulating forest ET and its components. The limited results were on a very short time scale, which neglects the effects of different climatic conditions on model accuracy.

Therefore, this study applied the SWH model in a subtropical plantation in southern China to estimate ET and partition ET into E and T. Furthermore, the modeled ET was verified by comparison with EC-observed ET, T by sap flow observations, and E by micro-lysimeter observations. We aimed to answer the following questions: (1) Is it feasible to use the SWH model in forests to estimate and partition ET? Would the results be reliable? (2) How would different climatic conditions affect the ET and the model results? To address this issue, 12 years of EC and corresponding environmental observation data were used to cover different climatic backgrounds. The findings of this study would help put forward the application of the SWH model. In addition, it would be helpful for improving the accuracy of estimation and separating ET. Based on the results, the controlling mechanisms of ET could be further explored.

## 2 Materials and methods

### 2.1 Site description

This study was conducted in a coniferous plantation at the National Qianyanzhou Critical Zone Observatory of Red Soil Hilly Region (QYZ) (26°44’29”N, 115°03’29”E, 102 m a. s. l.), which is a member of the Chinese Flux Observation and Research Network (ChinaFLUX). Coniferous trees were planted in this typical red soil hilly region in approximately 1985, with the dominant species of Masson pine (*Pinus massoniana* Lamb.), Slash pine (*Pinus elliottii* Englem.) and Chinese fir (*Cunninghamia lanceolata* Hook.). The plantation density was approximately 1460 trees ha^–1^. According to local climate records during 1989–2014, the mean annual temperature is 18.0 ± 0.4°C, and the annual precipitation is 1506.4 ± 306.1 mm. Further details of the QYZ site can be found in previous studies (Xu et al., 2017; Xu et al., 2021).

### 2.2 Observations and instrumentations

#### 2.2.1 Observations of environmental factors

The environmental factors were observed using sensors mounted on a 42 m iron tower. Above the land surface, seven level sensors were mounted to observe routine meteorological factors, such as air temperature, relative humidity, and wind speed. The sensors and environmental factors used in this study are listed as follows. Radiation was measured at a height of 41.6 m by a four-component net radiometer (Model CNR-1, Kipp & Zonen, Delft, The Netherlands) and a pyranometer (Model CM11, Kipp & Zonen). The air temperature and water vapor pressure were measured by a Model HMP45C sensor (Vaisala Inc., Helsinki, Finland) at 23.6 m. The soil water content was measured at depths of 5, 20 and 50 cm with TDR probes (Model CS615-L, Campbell Scientific Inc., Logan, UT, USA). The soil heat flux was measured by two heat flux plates (HFP01, Hukseflux Inc., Delft, The Netherlands) at a depth of 5 cm. Rainfall was monitored with a rain gauge (Model 52203, RM Young Inc., Traverse, MI, USA). These environmental variables were recorded with three CR10X dataloggers and a CR23X datalogger with a 25-channel solid-state multiplexer (Campbell Scientific Inc).

#### 2.2.2 Flux observations and data processing

The carbon and water fluxes were measured by an eddy covariance system, including a 3-D sonic anemometer (Model CSAT3, Campbell Scientific Inc.), an open-path CO_2_/H_2_O analyzer (Model LI-7500, Li-cor Inc., Lincoln, NE, USA) and a CR5000 datalogger (Campbell Scientific Inc.). All signals were collected at a frequency of 10 Hz, and the carbon and water fluxes were calculated and recorded at 30 min intervals. The flux data were processed using ChinaFLUX standard data processing methods (Yu et al., 2006; Xu et al., 2022; Zhu et al., 2023), including three-dimensional rotation (Zhu et al., 2005), Webb, Pearman and Leuning density correction (WPL correction) (Webb et al., 1980), storage calculations and spurious data removal. Data gaps were filled using the mean diurnal variation method, linear or nonlinear fitting or look-up table methods (Falge et al., 2001). The directly observed daytime and nighttime carbon fluxes are net ecosystem productivity (NEP) and ecosystem respiration (Re), respectively. Gross primary productivity (GPP) is a vital parameter in the SWH model. To obtain GPP, the Lloyd-Taylor equation was also used to extrapolate the daytime Re. NEP is the difference between GPP and Re, and then the GPP data can be determined (GPP=Re+NEP). The water flux equals the ecosystem evapotranspiration (ET).

#### 2.2.3 The observation of sap flow and calculation of transpiration

From May 2006 through April 2007, the sap flow velocity was observed using Granier-type thermal dissipation probes (model TDP-30, Dynamax Inc., Houston, TX, USA). The diameter of the probes is 1.2 mm with a length of 3 cm. The probes were installed in six trees comprising the dominant tree species of Masson pine, Slash pine and Chinese fir. The data were collected and recorded every 30 min by a datalogger (model SQ2040-4F16, Grant Instruments Ltd., Cambridgeshire, UK). The software provided by Dynamax was used to calculate the sap flux density (SFD, cm^3^ cm^–2^·h^–1^) by the following equation (Granier, 1985; Xu, 2011; Shen et al., 2015):


(1) $$SFD=3600\times 0.0119\times {\left(\frac{\Delta Tsap_{\max}}{\Delta Tsap}-1\right)}^{1.231}$$

where ΔTsap is the temperature difference measured between a constant heated needle and unheated needle. ΔTsap_max_ is the maximum temperature difference assessed during a period of zero flow, which usually appears at night or predawn. Granier (1985) and Lu et al. (2004) indicated that it is reasonable to confirm a ΔTsap_max_ every 7–10 days; hence, a ΔTsap_max_ was determined every 7 days in this study, and the SFD was calculated.

**Figure 1:**
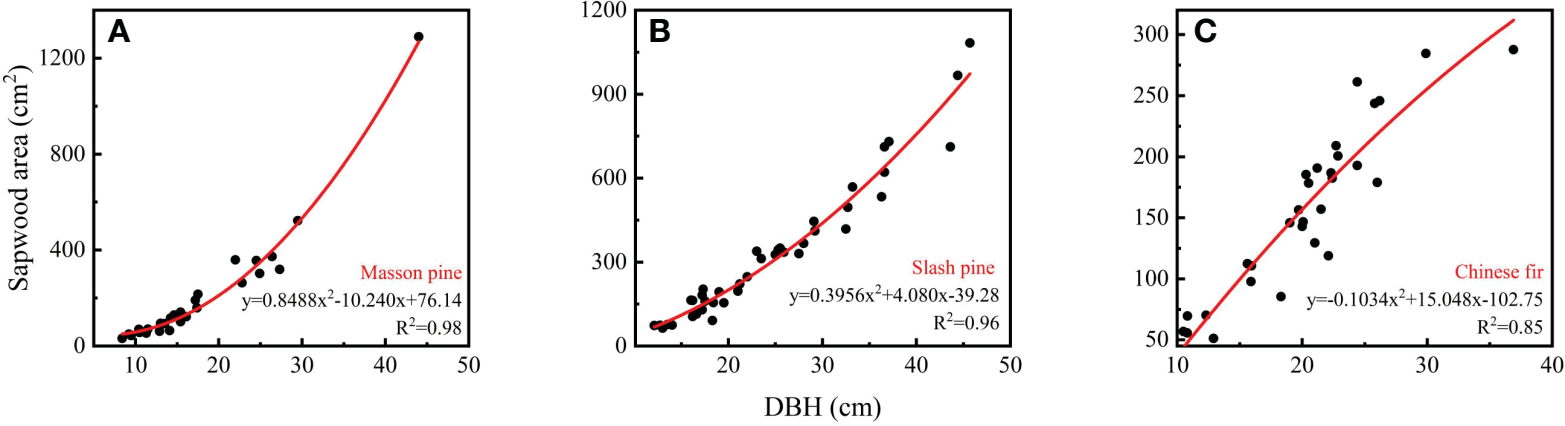
The relationship between sapwood area and diameter at breast height (DBH) for the three dominant tree species: **(A)** Masson pine; **(B)** Slash pine; **(C)** Chinese fir.

To calculate forest transpiration, two vital auxiliary data are needed. The first is the sapwood area of each tree, which is the active xylem that can transfer water. The second is the stand survey data, which provides detailed information on each tree in the plot. To clarify the area of each tree, more than thirty tree cores around the flux tower for each dominant tree species were collected to analyze the tree rings. According to the measurement of tree rings, the sapwood and heartwood could be separated, as the heartwood had a darker color. Then, the sapwood area could be calculated as follows:


(2) $$A=\pi \left({\left(D-d_{b}\right)}^{2}-D_{h}^{2}\right)$$

where A is the area of sapwood, D is the diameter of the tree sample, d_b_ is the depth of tree bark, and D_h_ is the diameter of the heartwood. According to the data obtained from the tree samples, nonlinear regressions were used to relate the sapwood area of the sampled trees to the diameter at breast height (DBH) for each species (**Figure 1**). Accordingly, the sapwood area for trees with different DBHs could be calculated accurately.

A 1 hm^2^ permanent plot around the flux tower was established in 2002, where a stand survey was conducted and the information for each tree was recorded. Combined with the stand survey data, the total sapwood area could be calculated, and then the transpiration of the plot could be calculated using the following equation:


(3) $$T=\sum_{1}^{i}\frac{{\text{SFD}}_{i}*A_{si}}{Ag}$$

where T is the measured forest transpiration (mm), SFD_i_ is the sap flux density of the given species, A_si_ is the total sapwood area of the given species, and A_g_ is the plot area.

#### 2.2.4 Observation of underlying surface evaporation

In this study, the homemade micro-lysimeter method was used to observe understory soil evaporation from July 2011 to October 2013. The micro-lysimeters were made of PVC with an inner diameter of 10 cm and a height of 15 cm. The micro-lysimeter was placed in a customized soil auger and punched into the soil until it was filled with undisturbed soil. Then, the soil auger and the micro-lysimeter were pulled out from the soil surface, and the micro-lysimeter was removed from the soil drill auger. The redundant soil at the bottom of the micro-lysimeter was removed, and the bottom was sealed with polyethylene tape. Then, the micro-lysimeter was weighed with an electronic balance with 1% accuracy. Around the flux tower, relatively flat plots under Masson pine, slash pine and Chinese fir stands were selected. Three micro-lysimeters were set up in each plot. The soil column in the micro-lysimeter was replaced every 3–5 days and was promptly replaced after a rainy day. The soil column and the micro-lysimeter were weighed every evening (18:00–19:00). The E was calculated according to the weight differences and the soil column surface area. The average of three replicates of three plots was calculated as the daily E of the understory soil.

### 2.3 Modeling methods

The SWH model is a two-source model that takes E and T into account as two separate sources (Hu et al., 2009; Hu et al., 2013). The model also considered the resistance for water vapor transfer, including the soil surface resistance (r_ss_, s m^−1^) existing between a depth where soil air is saturated with water vapor and the soil surface, resistance between soil surface to canopy height (r_as_, s m^−1^), resistance between the canopy to reference height (r_aa_, s m^−1^), stomatal resistance (r_sc,_ s m^−1^) existing between stomatal cavities and leaf surfaces, and aerodynamic resistance between leaf surface to canopy height (r_ac_, sm^−1^). The SWH model calculates the latent heat transferred by evapotranspiration λET as the sum of latent heat transferred by transpiration (λT) and evaporation (λE):


(4) $$\lambda \text{ET}=\lambda \text{T}+\lambda \text{E}={\text{C}}_{\text{c}}{\text{PM}}_{\text{c}}+{\text{C}}_{\text{s}}{\text{PM}}_{\text{s}}$$

(5) $${\text{PM}}_{\text{c}}=\frac{\Delta \text{R}+(C_{P}\text{VPD}-\Delta {\text{r}}_{\text{ac}}{\text{R}}_{\text{s}})/({\text{r}}_{\text{aa}}+{\text{r}}_{\text{ac}})}{\Delta +\gamma (1+({\text{r}}_{\text{sc}}/({\text{r}}_{\text{aa}}+{\text{r}}_{\text{ac}})))}$$

(6) $${\text{PM}}_{\text{s}}=\frac{\Delta \text{R}+(\text{ρ}C_{p}\text{VPD}-\Delta {\text{r}}_{\text{as}}(\text{R}-{\text{R}}_{\text{s}}))/({\text{r}}_{\text{aa}}+{\text{r}}_{\text{as}})}{\Delta +\gamma (1+({\text{r}}_{\text{ss}}/({\text{r}}_{\text{aa}}+{\text{r}}_{\text{as}})))}$$

where λ is the latent heat of vaporization and PM_c_ and PM_s_ represent the latent heat transferred by canopy transpiration and soil evaporation, respectively. C_c_ and C_s_ are the canopy resistance coefficient and soil surface resistance coefficient, respectively. Δ is the slope of the saturation vapor pressure versus temperature curve (kPa K^−1^). ρ is the density of air (1.293 kg m^−3^), and C_p_ is the specific heat at constant pressure (1012 J kg^−1^ K^–1^). VPD is the vapor pressure deficit (kPa), and γ is the psychrometric constant (0.067 kPa K^–1^). R and R_s_ (W m^–2^) represent the available energy input above the canopy and above the soil surface, respectively, and can be calculated as follows:


(7) R=Rn–G

(8) $${\text{R}}_{\text{s}}={\text{R}}_{\text{ns}}–\text{G}$$

where Rn and R_ns_ are the net radiation over the canopy and the understory net radiation (W m^–2^), respectively. G is the soil heat flux (W m^–2^). LAI is the leaf area index, and an 8-day LAI product based on the remote sensing technique was used in this study (https://modis.ornl.gov/sites/?id=cn_jiangxi_qianyanzhou_site2). The Savitzky–Golay filter was used to smooth out noise in LAI time-series data caused by cloud contamination and atmospheric variability. Furthermore, the cubic spline method was used to obtain the daily LAI data set to match the other data used in this study. R_ns_ can be estimated using the following equation:


(9) $${\text{R}}_{\text{ns}}=\text{Rn}*{\text{e}}^{-0.6\text{LAI}}$$

coefficients C_c_ and C_s_ were calculated as follows:


(10) $${\text{C}}_{\text{c}}=\frac{1}{1+({\text{ρ}}_{\text{c}}{\text{ρ}}_{\text{a}}/({\text{ρ}}_{\text{s}}({\text{ρ}}_{\text{c}}+{\text{ρ}}_{\text{a}})))}$$

(11) $${\text{C}}_{\text{s}}=\frac{1}{1+({\text{ρ}}_{\text{s}}{\text{ρ}}_{\text{a}}/({\text{ρ}}_{\text{c}}({\text{ρ}}_{\text{s}}+{\text{ρ}}_{\text{a}})))}$$

in which ρ_a_, ρ_c_ and ρ_s_ are calculated as:


(12) $${\text{ρ}}_{\text{a}}=(\Delta +\gamma ){\text{r}}_{\text{aa}}$$

(13) $${\text{ρ}}_{\text{c}}=(\Delta +\gamma ){\text{r}}_{\text{ac}}+\gamma {\text{r}}_{\text{sc}}$$

(14) $${\text{ρ}}_{\text{s}}=(\Delta +\gamma ){\text{r}}_{\text{as}}+\gamma {\text{r}}_{\text{ss}}$$

The three aerodynamic resistances r_ac_, r_as_ and r_aa_ were calculated using the same approach as Shuttleworth and Wallace (1985). The soil surface resistance r_ss_ was estimated as a function of the soil water content (Lin and Sun, 1983):


(15) $${\text{r}}_{\text{ss}}={\text{b}}_{1}{\left(\frac{\text{SMC}}{\text{SWC}5}\right)}^{{\text{b}}_{2}}+{\text{b}}_{3}$$

where SMC and SWC5 are the saturated soil water content and soil water content at a depth of 5 cm (m^3^ m^–3^), respectively, and b_1_, b_2_, and b_3_ are empirical constants. An improved Ball-Berry model (Ball et al., 1987) that considered the effects of soil moisture was used to estimate r_sc_:


(16) $${\text{r}}_{\text{sc}}=\frac{1}{{\text{g}}_{0}+{\text{a}}_{1}\text{f}(\text{SWC}5){\text{P}}_{\text{n}}\text{RH}/{\text{C}}_{\text{s}}}$$

(17) $$\text{f}(\text{SWC}5)=\frac{\text{SWC}5–\text{WP}}{\text{FWC}–\text{WP}}$$

where g_0_ and a_1_ are empirical parameters, FWC and WP are field capacity and the wilting point, respectively, and P_n_ (μmol m^2^ s^–1^) is the photosynthetic rate, which was replaced by GPP in this study. RH is the relative humidity, and C_s_ is the leaf surface CO_2_ content (ppm). FWC and WP were instead by the maximum and minimum soil water contents observed at a depth of 5 cm, respectively. The CO_2_ concentration above the canopy measured by the open-path CO_2_/H_2_O gas analyzer was used as C_s_, and g0 was assigned as a value near zero (0.00001).

## 3 Results

### 3.1 Seasonal and interannual variations in environmental factors and evapotranspiration

**Figure 2:**
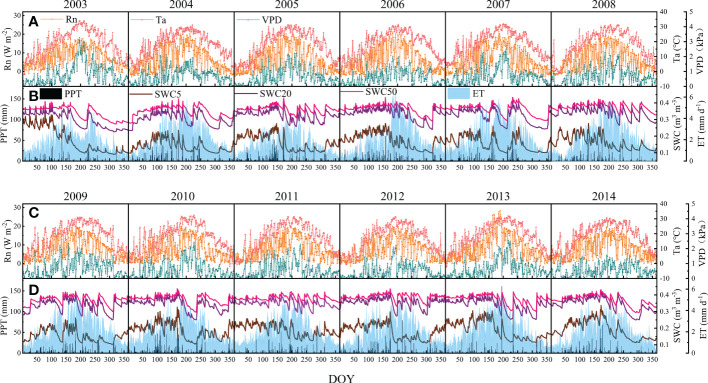
Seasonal and interannual variations in environmental factors and ET at the QYZ station from 2003 to 2014: **(A)** net radiation (Rn), air temperature (Ta) and vapor pressure deficit (VPD) from 2003 to 2008; **(B)** precipitation (PPT), soil water content at 5 cm (SWC5), soil water content at 20 cm (SWC20), soil water content at 50 cm (SWC50) and ET from 2003 to 2008; **(C)** Rn, Ta and VPD from 2009 to 2014; **(D)** PPT, SWC5, SWC20, SWC50 and ET from 2009 to 2014.

**Figure 3:**
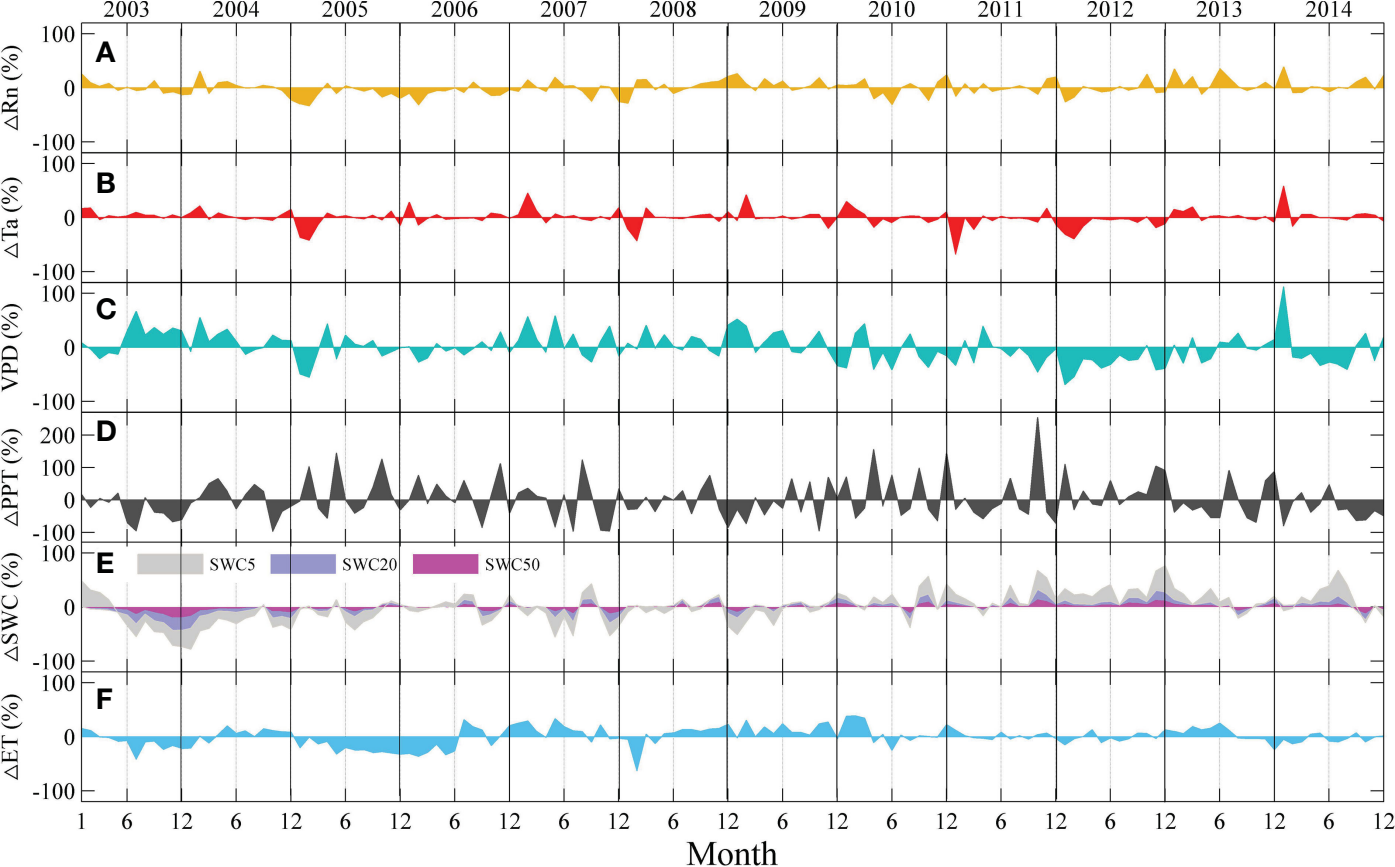
Seasonal and interannual anomalies in environmental factors and evapotranspiration (ET) at the QYZ station from 2003 to 2014: **(A)** net radiation (Rn); **(B)** air temperature (Ta); **(C)** vapor pressure deficit (VPD); **(D)** precipitation (PPT); **(E)** soil water content at 5 cm (SWC5), soil water content at 20 cm (SWC20), soil water content at 50 cm (SWC50); **(F)** ET.

Environmental factors, including net radiation (Rn), air temperature (Ta), vapor pressure deficit (VPD), precipitation (PPT) and soil water contents at depths of 5, 20, and 50 cm (SWC5, SWC20, and SWC50, respectively), are expected to be the dominant environmental factors for variations in ET; hence, their seasonal and interannual variations and anomalies together with ET are presented in **Figures 2**, **3**. The seasonal patterns of Rn, Ta and VPD were consistent, following unimodal patterns with peaks in July or August. It rained more in the first half year, and hence, the PPT was higher. The SWCs were greatly affected by PPT. Therefore, the SWCs were higher in the first half year and decreased in July. The ET generally showed a unimodal variation with a peak in July but showed great interannual variability.

During the study period, the climatic conditions varied across years (**Figure 3**). The abnormal climatic events could mainly be categorized into two types: cold-spring years with greater negative Ta anomalies in the early spring (January–March) and summer-drought years with lower PPT in the summer or autumn. Negative Ta anomalies were found in the early springs of 2005, 2008, 2011 and 2012. Among them, the lower Ta in 2005 lasted for the longest time with lower Rn and VPD. Negative PPT occurred frequently in summer and autumn in this region but with different distribution characteristics. In 2003, obvious negative anomalies in PPT induced great decreases in SWCs in the second half of the year. However, in other years, such as 2007, 2013, and 2014, although negative PPT anomalies were found, they did not cause great decreases in SWCs. In addition, the occurrence time of PPT shortage should be noted. In 2007, the PPT shortage occurred at the end of the year, which would have weaker effects on the ecosystem. The anomalies in Rn were relatively small, but they showed obvious negative anomalies in 2005, and positive anomalies were found in the first half year in 2013 and 2014. For the observed ET, obvious negative values were found in the second half of 2003 and throughout 2005.

### 3.2 The modeling results of evapotranspiration

**Figure 4:**
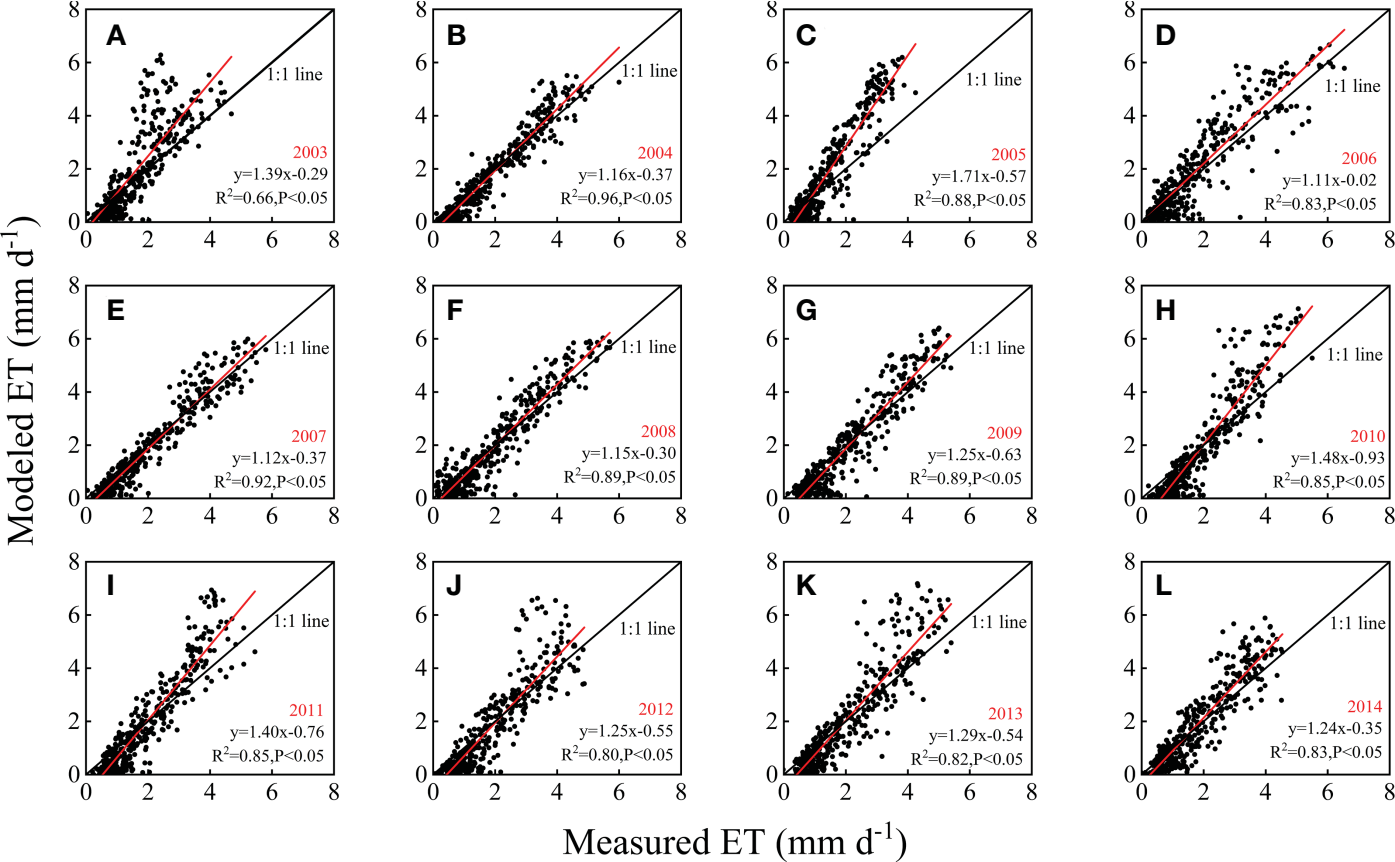
The relations between measured evapotranspiration (ET) and modeled ET based on daily scale data from 2003 to 2014. The solid line is the 1:1 line, and R^2^ and P indicate the determination coefficient and P value, respectively. **(A)** 2003; **(B)** 2004; **(C)** 2005; **(D)** 2006; **(E)** 2007; **(F)** 2008; **(G)** 2009; **(H)** 2010; **(I)** 2011; **(J)** 2012; **(K)** 2013; **(L)** 2014.

**Figure 5:**
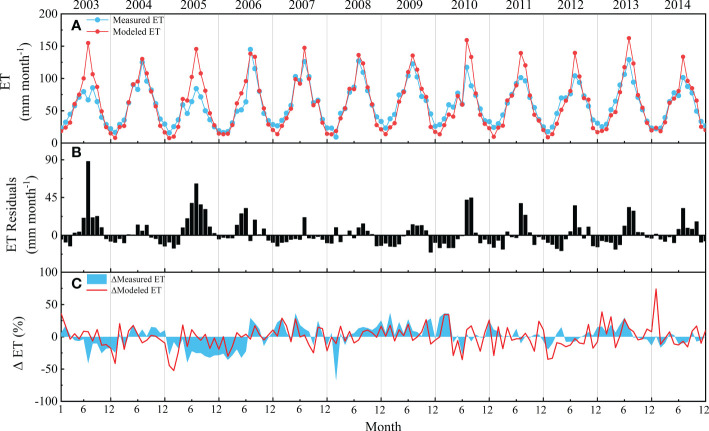
Comparison of measured evapotranspiration (ET) and modeled ET: **(A)** seasonal and interannual variations in measured ET and modeled ET; **(B)** seasonal and interannual variation in ET residuals; **(C)** seasonal and interannual anomalies in measured ET and modeled ET.

Generally, the modeled ET was closely related to the ET measured by the EC system and could track seasonal variations in ET (**Figures 4**, **5**). The slopes of the linear fitting equations for the modeled and measured ET ranged from 1.11 to 1.71 with high determinate coefficients. In most of the years, the R^2^ values were higher than 0.80, with an average of 0.85, which indicated that it would be feasible and reliable to use the SWH model to estimate ET in this subtropical forest. To give a sharp picture of model performances, in addition to the linear fitting lines, the 1:1 line was also drawn in **Figure 4**. The model performed best in 2004 and 2007, with slopes of the fitting line of 1.16 and 1.12, R^2^ values of 0.96 and 0.92, respectively, and the smallest residuals of the model (**Figures 4B**, **5B**). In 2006, the slope of the fitting line was closest to 1.0, when Rn, Ta, PPT and SWC were all close to the multiyear averages and there were no abnormal climate events (**Figures 2**, **3**), but the model residuals were larger in April to June. In the other years, the modeled ET was generally higher than the EC-observed ET. In the years with extreme climatic events, the model greatly overestimated ET. For instance, in 2005, a long-term lower Ta was found in early spring, and the modeled ET was much higher than the observed values (**Figure 4C**). In the summer-drought year of 2003, similar overestimation results were found (**Figure 4A**). Since 2010, the model has overestimated ET, especially in summer. Consequently, the slopes were larger.

According to the seasonal dynamics of ET (**Figure 5A**), the modeled and observed ET showed similar seasonal patterns. However, overestimations were often found during May–October with higher residuals, especially in 2003 and 2005 (**Figure 5B**). The model was relatively weak in tracking the interannual variabilities, which could be found by comparing the anomalies of modeled and measured ET (**Figure 5C**). According to the annual accumulated values, the model underestimated the interannual variations to some extent, with a coefficient of variation (CV) of 4.2%, which was much lower than the measured CV of 10.2% (**Table 1**). However, the model overestimated the interannual variations in the beginning of the year with higher CV, as it sometimes overestimated the anomalies caused by anomalies in Rn, such as in the beginning of 2014 (**Figure 5C**).

**Table 1:** Multiyear monthly averages and coefficient of variation (CV) of measured evapotranspiration (ET) and modeled ET.

Month	Measured ET (mm)	CV (%)	Modeled ET (mm)	CV (%)
1	21.8	19.0	13.5	34.2
2	27.5	28.9	20.6	26.3
3	43.6	16.5	32.8	19.0
4	60.8	10.9	57.7	13.6
5	75.8	19.9	77.4	12.3
6	82.2	18.2	92.2	15.9
7	112.5	18.5	143.5	7.0
8	95.2	11.7	114.0	10.5
9	71.5	12.3	78.0	10.0
10	53.3	16.9	56.4	16.2
11	35.1	13.8	27.9	11.7
12	26.7	16.0	18.1	15.0
Annual	705.8	10.2	732.0	4.2

### 3.3 Separation of evapotranspiration into transpiration and evaporation and the verifications

**Figure 6:**
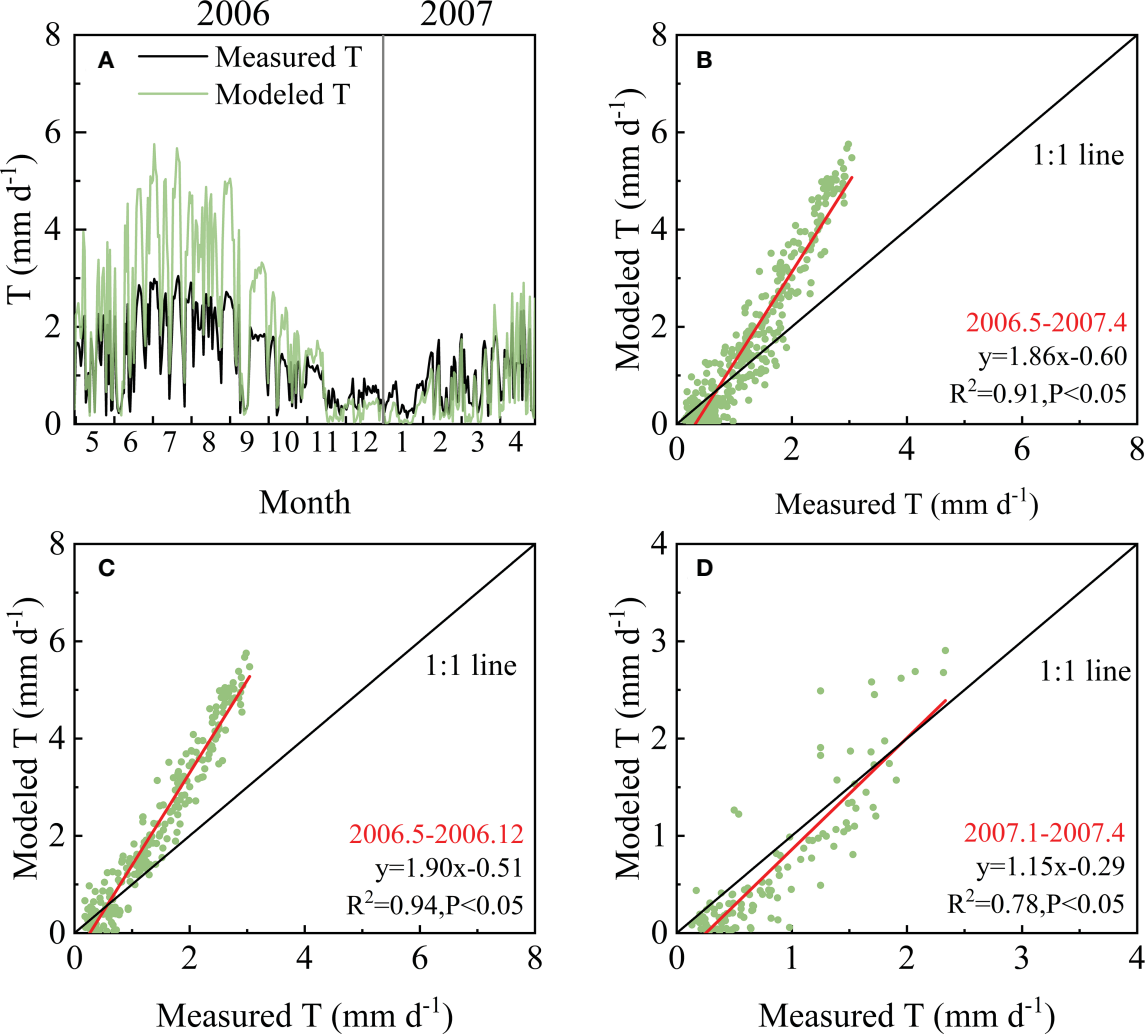
Comparison of measured and modeled transpiration (T) from May 2006 to April 2007: **(A)** Seasonal variation in measured and modeled T from May 2006 to April 2007; **(B)** the relation between measured and modeled T analyzed based on all data; **(C)** the relation between measured and modeled T during May 2006-December 2006; **(D)** the relation between measured and modeled T during January 2007-April 2007.

The ET was separated into transpiration (T) and evaporation (E) by the SWH model. The separation results were verified using T measured by the sap flow technique and E measured by the micro-lysimeter method. The modeled T showed a consistent seasonal pattern with the measured T and was significantly related to the observed T with an R^2^ of 0.91 (**Figure 6**). However, the slope of the fitting line was as high as 1.86, indicating that the modeled T was higher than the measured values, especially in peak seasons. In the dormant season, the values of modeled and measured T were closer, and the slope of the linear fitting equation was 1.15 (**Figure 6D**).

**Figure 7:**
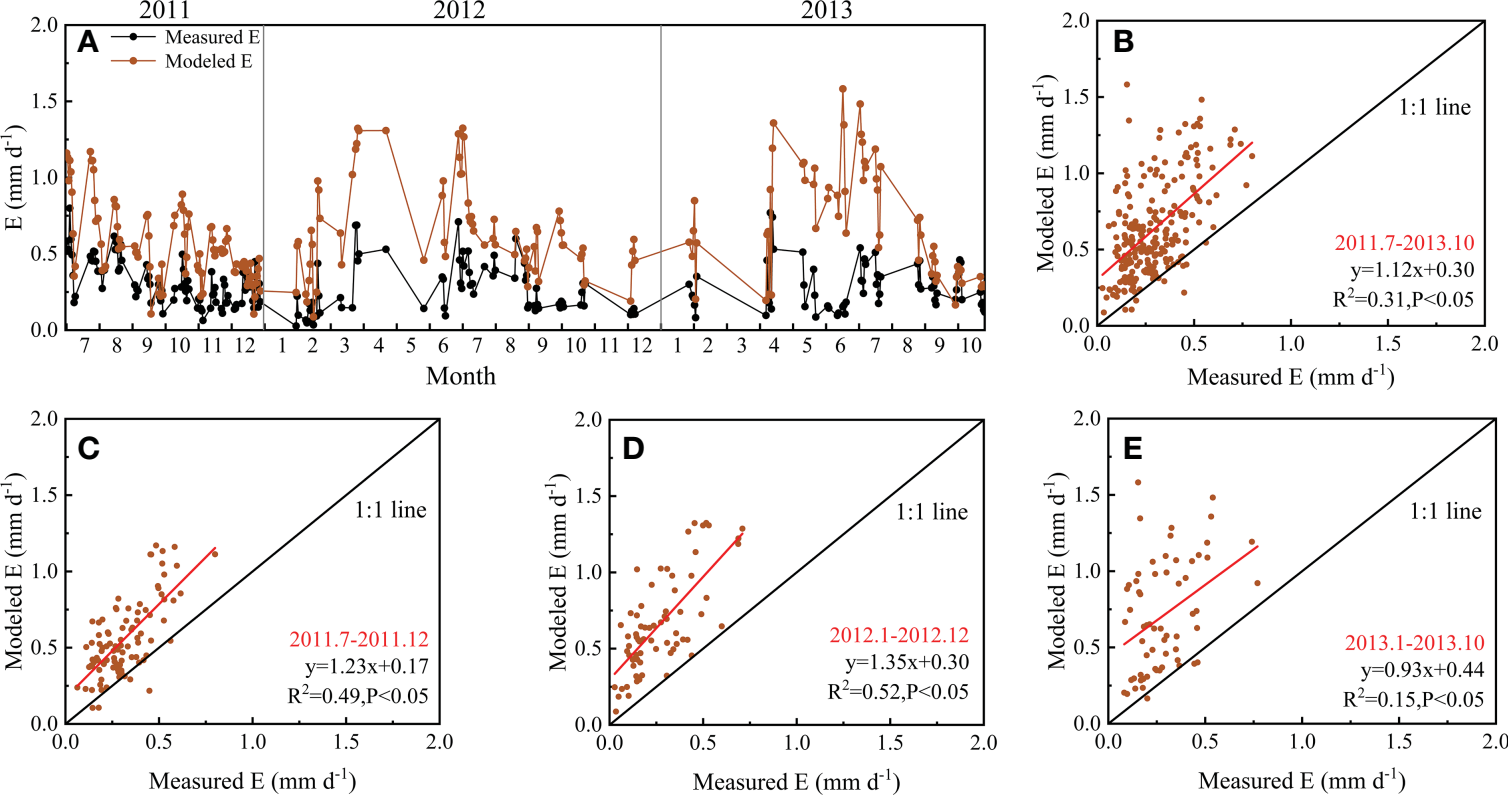
Comparison of measured and modeled evaporation (E) from July 2011 to October 2013: **(A)** Seasonal variation in measured and modeled E from July 2011 to October 2013; **(B)** the relation between measured and modeled E analyzed based on all data; **(C)** the relation between measured and modeled E in 2011; **(D)** the relation between measured and modeled E in 2012; **(E)** the relation between measured and modeled E in 2013.

More than two years of observed E were used to verify the model-separated E (**Figure 7**). The model could track the seasonal variations in E but also overestimated E. The observation began in July 2011, in which year, E was observed more frequently. The fitting line for modeled and measured values was closer to the 1:1 line in 2011. In 2012, the model overestimated E during April–June, when it rained more often (**Figure 2**). In 2013, the frequency of E observations was limited due to frequent rain, and the relations between the modeled and measured values were not as close as in 2011. It was also found that the modeled values were higher than the observed values, especially in wet seasons.

**Figure 8:**
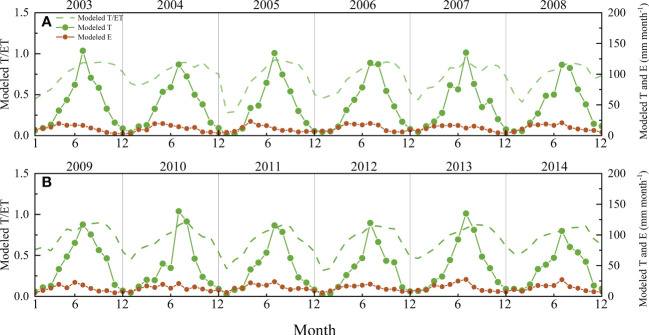
Seasonal and interannual variation in the ratio of modeled transpiration (T) to modeled evapotranspiration (ET), modeled T, and modeled evaporation (E) from 2003 to 2014: **(A)** 2003-2008 and **(B)** 2009-2014.

The SWH model could successfully partition ET into E and T with obvious seasonal and interannual variability (**Figure 8**). During the study period, the average value of T/ET was 0.71, with a maximum of 0.92 and a minimum of 0.28. The E/ET ranged from 0.08 to 0.72, with an average of 0.29. The T/ET generally peaked in June or July, but it peaked in different periods with great discrepancy across years.

## 4 Discussion

### 4.1 The effects of climate background on ET and the modeling results

Currently, along with advancing observation techniques, the parameters requested by the models are much easier to obtain (Lian et al., 2018; Gong et al., 2020; Yan et al., 2022). Consequently, a much wider application of ET models is expected, and models that have been verified and have good performance could be put to wider use. In this study, the SWH model was used to model ET over a long time scale, and ET measured by the EC system was used to verify the model results. According to the multiyear average, the SWH model only overestimated 26.2 mm on the annual scale (**Table 1**), which was only 3.7% higher than the measured ET. The performance is comparable with and even better than other models (Gao et al., 2016), indicating that the SWH model could be well applied in forest ecosystems. Moreover, SWH could partition the ET, which would be beneficial for further studies.

**Figure 9:**
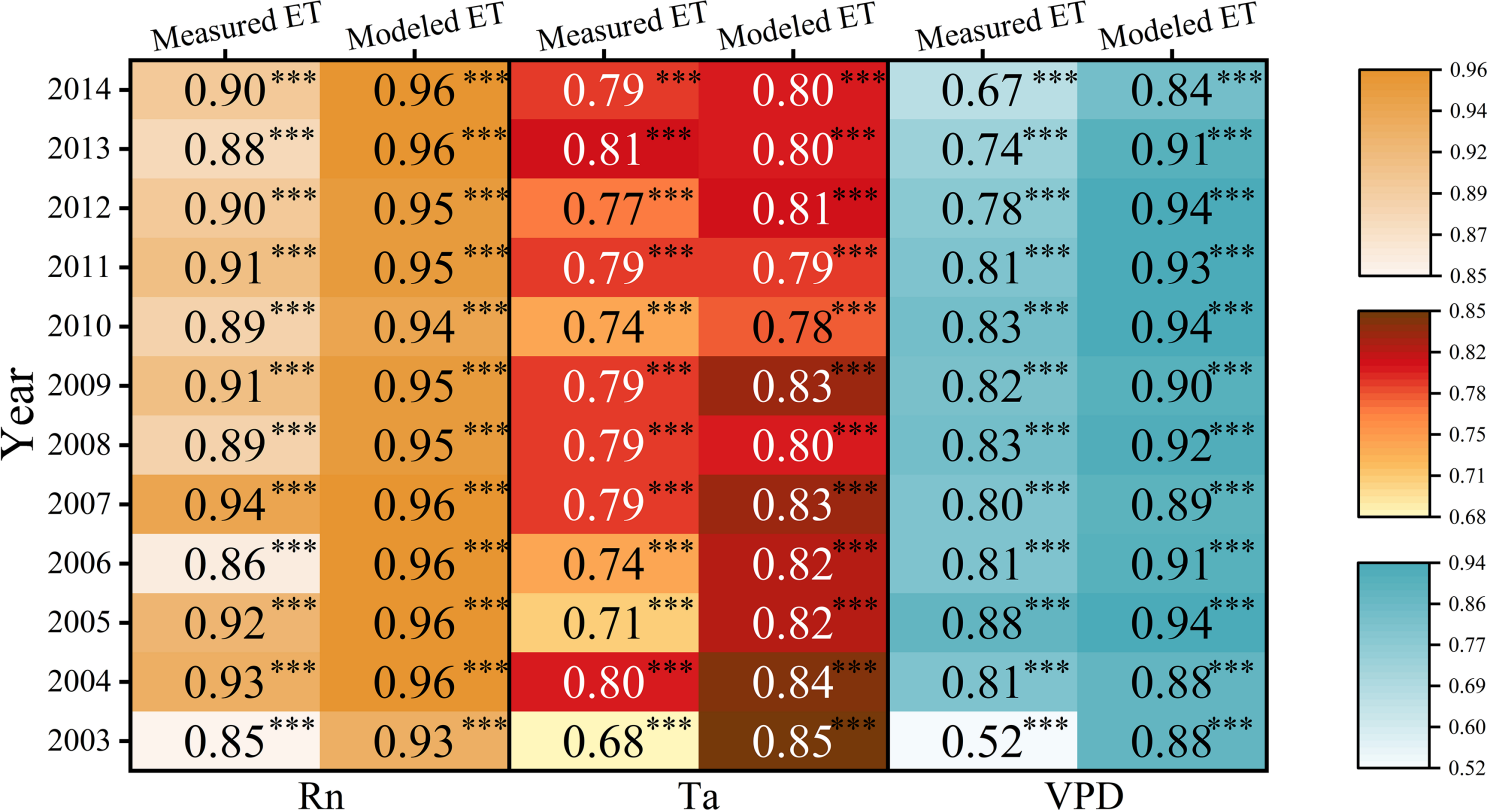
Responses of measured and modeled evapotranspiration (ET) to net radiation (Rn), air temperature (Ta) and vapor pressure deficit (VPD). Darker colors indicate stronger relations between measured or modeled ET and Rn, Ta, and VPD. The three-star asterisk indicates P<0.001.

This study took advantage of long-term ET observations, based on which the effects of different climatic conditions on the model performance could be discussed specifically (**Figures 4**, **5**). The model performed best in 2004 and 2007, in which year the modeled ET was closely related to the measured ET and perfectly followed the seasonal variations. The good performance could be attributed to the relations between the measured ET and Rn, when Rn showed the strongest control on ET (**Figure 9**). In addition, there were no long-term climatic anomalies in these two years. In 2004, the climatic factors were close to the multiyear average, with small anomalies in Rn and Ta. The higher PPT in the beginning of the year exactly mediated the drought caused by the PPT shortage in late 2003 (Wen et al., 2010). In 2007, although it met the shortage of PPT in July, the PPT in August alleviated the drought. The shortage of PPT was also found in October to November, but the drought in the dormant season had limited instant influences on the ecosystem. In addition to these two years, the model was expected to perform well in 2006. In 2006, the heat and water resources matched well without large anomalies in the key environmental factors, and hence, the productivities were higher according to previous studies (Xu et al., 2017; Zhang et al., 2018). However, although with the expected parameters of the fitting equation (y=1.11x–0.02), the model residuals in 2006 were relatively larger. In contrast to other years, in which the model mainly overestimated ET in the periods with lushest vegetations the most vegetation (July–August), the overestimation of ET occurred during April–June in 2006. This might be ascribed to the legacy effects of low temperature in 2005 (Xu et al., 2014). The trees might sprout fewer new branches in 2005, and hence, the trees would be less lush in the beginning of 2006, as the needles of the dominant coniferous trees are generally biennials. Therefore, in the real world, ET would be limited during the first half of 2006 (**Figure 5C**), after which the new sprout needles began to be active. However, the model could not track this variability, and hence, larger model residuals in April–June were found when the T of old needles accounted for a larger portion of ET. This is reasonable, as previous studies have indicated the legacy effects of climatic factors (Richardson et al., 2009; Xu et al., 2014; Zona et al., 2014). The results indicated that the model would perform well in normal years but also indicated the weakness of the model in tracking the legacy effects of environmental and biological variabilities.

The performance of the model was not good in 2003 and 2005. The fitting lines were relatively far from the 1:1 line (**Figures 4A, C**). The large residuals lasted for more than five months, and the annual accumulated residuals were largest among the twelve years, with the sum of the absolute values of the residuals being more than 200 mm (**Figure 5B**). The year 2003 was the most drought year during the study period, with the most severe PPT shortage and largest negative anomalies in SWCs in the second half year (**Figures 2**, **3**). Consequently, the shortage of available water supply would directly induce decreases in ET. In addition, extreme drought induces irreversible effects on ecosystems, such as the earlier fall of needles or wither of understory vegetation. These ecosystem responses were supposed to have more important effects on ET (Xu et al., 2014), whereas climatic factors showed relatively weaker effects on ET with relatively smaller correlation coefficients (**Figure 9**). However, the model could not track the ecosystem responses, and the modeled ET was still highly dependent on climatic factors, which could be inferred from the higher correlation coefficients between modeled ET and environmental factors. Consequently, the modeled ET was much higher than the measured ET. Similarly, the longest lower Ta in the beginning of 2005 (**Figure 2**) induced the postponement of the phenology (Xu et al., 2017), and probably the legacy effects of low temperature led to the larger negative anomalies in measured ET and lasted for the whole year, even limiting the ET in the first half year of 2006. However, the model amplified the instant effects of lower Ta, which was embodied in the negative anomalies in January to March in 2005, and it could not take the legacy effects of low Ta into account, hence greatly overestimating ET since April in 2005. These legacy effects have also been indicated by previous studies through time lag effect analysis (Xu et al., 2014; Xu et al., 2017).

In other years, the models performed relatively well, but they have overestimated ET in summer since 2010. This could be ascribed to the stronger water retention effects accompanied by forest development (Xu et al., 2018), which would induce less dependency of ET on VPD. It could be evidenced by the observation data, which indicated that the VPD increased since 2010, and the measured ET was less and less related to VPD (**Figure 9**). This would be reasonable, as the VPD works as a pull force for ET. When the VPD decreased since the microclimate became more humid in the forest, the control effects of VPD on ET would not be as strong as before. However, the modeled ET was still highly related to VPD. The forest in the real world responds to the changing microclimate but not the modeled forest. The VPD was higher in peak seasons, which may be one of the reasons the ET was overestimated by the SWH model in peak seasons.

The anomalies in the modeled ET are quite sensitive to short-term climatic anomalies. For instance, Rn was higher in January 2014, and obvious positive anomalies in modeled ET were found. However, the measured ET did not respond to the short-term higher Rn. This phenomenon indicated that the model could track but overestimate the instant effects of climatic factors, while the ecosystem had buffer effects for short-term fluctuations in climatic factors. As previously indicated, climatic factors drove the seasonal pattern of ET, but ecosystem responses played the dominant role in the interannual variabilities in ET (Xu et al., 2014; Wu et al., 2015; del Campo et al., 2019).

The SWH model could be used to model ET in subtropical forests, and it performed well in most years without abnormal climatic events. The modeled ET could track the seasonal variations in ET, but it was weaker in accurately estimating the interannual variability. Models can track or amplify the effects of meteorological factors that instantly and directly affect ET, but they cannot track the legacy effects of environmental factors. This study analyzed the specific performances of the SWH model across different climatic years based on long-term data. The following important implications could be inferred from the detailed analyses conducted in this study. First, the model could be further improved by adjusting parameters according to different climatic years (such as cold-spring years and summer-drought years). In addition, the changing dependence of ET on environmental factors should be taken into serious consideration, and the parameters should be adjusted even when the same model is applied in the same ecosystem for a relatively long term, as the ecosystem is a dynamic system that changes over time. Therefore, further and deep studies on model application over large spatial and temporal scales are still needed, which would help improve the understanding of the model results and hence modify the models to make them more accurate.

### 4.2 Partitioning of evapotranspiration and the verifications

Partitioning ET and clarifying the ratio between E or T to ET has received wide attention (Zhu et al., 2015; Lian et al., 2018; Bhattarai et al., 2022; Cao et al., 2022; Chen et al., 2022). The SWH method performs very well in partitioning ET into transpiration and evaporation and has been widely applied in crop and grassland ecosystems (Hu et al., 2013; Zhao et al., 2020; Bao et al., 2021b). However, relatively few studies have been conducted in forests, as forests have complicated vertical structures. If the partitioning results could be proven to be reliable, it would help ecologists better understand the underlying mechanisms of variations in the ET of forests (Niu et al., 2019). Therefore, we conducted *in situ* experiments at the QYZ station and compared the model results with the measurements.

The partitioning results showed that the modeled T was significantly related to the measured T and could track the seasonal dynamics of the measured T obtained through the sap flow and scale-up method (**Figure 6**), which indicated that it is feasible to use this model in forests. During the observation period (May 2006–April 2007), the average value of T/ET according to the model was 0.67, whereas it was 0.60 according to the measured T/ET. This ratio is consistent with previous studies, indicating that the T/ET in forests varied in the range of 0.50–0.79 (Fatichi and Pappas, 2017). However, when comparing the modeled T to measured values, it seemed that the model overestimated T to some extent, especially in the peak season. During May–October in 2006, the modeled T accounted for 83.3% of ET on average, with a range from 47.4% to 92.0%. Meanwhile, the measured T accounted for 65.1% of the measured ET on average, with a range from 12.4% to 100%. According to our results, during this period, the SWH model overestimated daily ET by an average of 0.4 mm d^–1^ (15.6% higher than measured ET) but overestimated daily T by an average of 1.0 mm d^–1^ (63.1% higher than measured T). That would be unreasonable; hence, this discrepancy must be from the overestimation of the model and underestimation induced by scaling up sap flow measurements to forest transpiration.

As the absolute true T of the forests could not be measured, the T acquired through different methods are expected to show the same seasonal pattern and similar responses to environmental factors, which could be used to verify each other or as supplementary data. The sap flow technique based on thermal sensors involves some bias according to previous studies (Flo et al., 2019; Dix and Aubrey, 2021). In addition, the limited sample size induced by tree mortality and instrument damage may also induce some estimation bias. Moreover, it is not easy to scale up stand T to forest T, as considerable auxiliary data are needed. Fortunately, at this site, the forest was an artificial coniferous forest, and hence, the tree communities were relatively homogeneous. In addition, there is a permanent large plot in QYZ, where every tree in the plot was numbered and measured. Combined with the tree-ring samples collected around the plot, the sapwood area could be estimated relatively accurately. Therefore, it provided the possibility to evaluate the performance of the SWH model. However, there are some broadleaf species and understory shrubs induced by the natural succession process, whose T was neglected when we scaled up the sap flow measurements to forest T. In addition, the water and heat resources were abundant in 2006; hence, the understory vegetation may contribute more to forest T than in other years (Xu et al., 2017). This may be why the relatively larger differences between measured and modeled T appeared in peak seasons. Dynamic ecosystem responses to changing environmental factors are the main cause of interannual variability and the most difficult issue for models (Xu et al., 2014; MacBean et al., 2021). Meanwhile, suitable environmental factors would make the SWH model overestimate T to some extent, as the model would be more dependent on climatic factors. Although the SWH model may overestimate T, the amplitude is acceptable (Lawrence et al., 2007; Nguyen et al., 2021). The results of the measured and modeled T could validate each other in seasonal variations, and their values both fell in a reasonable scope, which indicated that the SWH model was suitable for partitioning ET in forests. However, more studies based on longer time scales and in other regions could be further conducted to improve the accuracy of the partitioning effects.

The E modeled by SWH showed consistent variation patterns with measured E during the observation period (**Figure 7**), and the values are closely related, indicating that it is feasible to use the model to estimate E in the forest. The average value of modeled E/ET was 0.37, 0.39, and 0.32 in the observed years of 2011, 2012 and 2013, respectively, but the corresponding measured E/ET was 0.17, 0.11, and 0.11. Micro-lysimeter measurements were conducted more frequently in 2011 but much less frequently in 2012 and 2013. The undisturbed soil column in micro-lysimeters could not be changed too often. Therefore, the soil column would not be changed when it rained slightly on given days, as sometimes the light rain did not go through the canopy. However, it would underestimate E sometimes. This might be one reason why the relation between modeled and measured E was not as good as expected. The results verified that the SWH model could be used to estimate E, although it might slightly overestimate E. Based on the acceptable partitioning results (**Figure 8**), the underlying mechanisms of ET variabilities could be explored based on a clearer understanding of variations in E and T. For example, it is obvious that T generally increased sharply after May and dropped sharply after October in this forest, and the ratio of T and ET varied greatly among years. Some special phenomena could also be detected according to the partition results. For instance, in 2005, the T/ET was very low at the beginning of the year, and the T/ET peaked later in 2005 than in other years. Consequently, the wider use of the SWH model on a larger scale would help further studies on ET.

Based on the modeled values and *in situ* measurements, the modeled E and T were found to have consistent seasonal patterns with the measured E and T, respectively (**Figures 6**, **7**). The data measured using different methods and from different scales matched each other and could be cross validated, although they have some discrepancies. The *in situ* E measurements probably underestimated E slightly due to the limited samples and limited water supplementation from the surrounding soils. The *in situ* sap flow underestimated T because only the dominant trees were measured, and the transpiration from other trees, shrubs, and grasses was neglected (Xu, 2011). In addition, T would be underestimated to some extent when the scale-up calculations were conducted in combination with the stand investigation data. Compared with previous studies (Zhu et al., 2015; Ren et al., 2019), the modeled results fell in the acceptable range, although they may overestimate T or E to some extent. However, these discrepancies were inevitable and reasonable, and the measured and modeled E (T) values are useful in validating each other, as they have reasonable values and show consistent variation patterns. In summary, the SWH model performed well at partitioning ET in this subtropical forest and could be further optimized according to its performance at different climatic backgrounds. Since the absolute true E and T could not be obtained in forests, the improvements and wide use of the models would be useful in studying forest water processes under global climate change.

## Data availability statement

The original contributions presented in the study are included in the article/supplementary material. Further inquiries can be directed to the corresponding authors.

## Author contributions

MX, TZ, and HW conceived and designed this study. MX, TZ, QM, SL, YT, and QW collected and analyzed the data, drew the figures, and wrote the manuscript. MX, FY, FX, BY, HZ, and SZ conducted the field observations and investigations. All authors contributed to the article and approved the submitted version.
